# Metabolic features of recurrent major depressive disorder in remission, and the risk of future recurrence

**DOI:** 10.1038/s41398-020-01182-w

**Published:** 2021-01-11

**Authors:** Roel J. T. Mocking, Jane C. Naviaux, Kefeng Li, Lin Wang, Jonathan M. Monk, A. Taylor Bright, Caroline A. Figueroa, Aart H. Schene, Henricus G. Ruhé, Johanna Assies, Robert K. Naviaux

**Affiliations:** 1 Department of Psychiatry, Amsterdam UMC, Academic Medical Center, University of Amsterdam, Meibergdreef 5, 1105 AZ Amsterdam, The Netherlands; 2 The Mitochondrial and Metabolic Disease Center, University of California, San Diego School of Medicine, 214 Dickinson St., Bldg CTF, Rm C107, San Diego, CA 92103-8467 USA; 3 Department of Neurosciences, University of California, San Diego School of Medicine, 214 Dickinson St., Bldg CTF, Rm C107, San Diego, CA 92103-8467 USA; 4 Department of Medicine, University of California, San Diego School of Medicine, 214 Dickinson St., Bldg CTF, Rm C107, San Diego, CA 92103-8467 USA; 5 Department of Psychiatry, Radboud University Medical Center, Nijmegen, the Netherlands; 6 Donders Institute for Brain, Cognition and Behavior, Radboud University Nijmegen, Nijmegen, the Netherlands; 7 Department of Pediatrics, University of California, San Diego School of Medicine, 214 Dickinson St., Bldg CTF, Rm C107, San Diego, CA 92103-8467 USA; 8 Department of Pathology, University of California, San Diego School of Medicine, 214 Dickinson St., Bldg CTF, Rm C107, San Diego, CA 92103-8467 USA; 9 Present Address: Colt Neck Labs, 838 E High St 202., Lexington, KY 40503 USA; 10 Present Address: School of Social Welfare, University of California, Berkeley, CA 94720 USA

**Keywords:** Predictive markers, Depression

## Abstract

Recurrent major depressive disorder (rMDD) is a relapsing-remitting disease with high morbidity and a 5-year risk of recurrence of up to 80%. This was a prospective pilot study to examine the potential diagnostic and prognostic value of targeted plasma metabolomics in the care of patients with rMDD in remission. We used an established LC-MS/MS platform to measure 399 metabolites in 68 subjects with rMDD (*n* = 45 females and 23 males*)* in antidepressant-free remission and 59 age- and sex-matched controls (*n* = 40 females and 19 males). Patients were then followed prospectively for 2.5 years. Metabolomics explained up to 43% of the phenotypic variance. The strongest biomarkers were gender specific. 80% of the metabolic predictors of recurrence in both males and females belonged to 6 pathways: (1) phospholipids, (2) sphingomyelins, (3) glycosphingolipids, (4) eicosanoids, (5) microbiome, and (6) purines. These changes traced to altered mitochondrial regulation of cellular redox, signaling, energy, and lipid metabolism. Metabolomics identified a chemical endophenotype that could be used to stratify rrMDD patients at greatest risk for recurrence with an accuracy over 0.90 (95%CI = 0.69–1.0). Power calculations suggest that a validation study of at least 198 females and 198 males (99 cases and 99 controls each) will be needed to confirm these results. Although a small study, these results are the first to show the potential utility of metabolomics in assisting with the important clinical challenge of prospectively identifying the patients at greatest risk of recurrence of a depressive episode and those who are at lower risk.

## Introduction

Major depressive disorder (MDD) affects 16.1 million US adults and costs $210 billion annually^1^. MDD’s worldwide point prevalence is 6%^2^. Recurrence risk after a first MDD-episode is 3–6 times the background population risk^3^, with most patients having a recurring-remitting course with five lifetime episodes on average. This lifelong recurrence risk accounts substantially to the overall burden of MDD^4^ and to the risk of suicide^5^. If we could better understand molecular bases of recurrent major depressive disorder (rMDD), we might develop prospective risk markers and novel targets for prevention.

Standard clinical variables moderately predict recurrence risk^4^ but more robust biomarkers are needed. Evidence for psychological theories of MDD-recurrence is limited and has not yet resulted in risk prediction tools that have been included in clinical guidelines^6^. Several biological pathways have been related to MDD in general, but only a limited number of studies specifically investigated rMDD. A recent meta-analysis comprehensively reviewed all evidence on biological factors predicting recurrence, including neuroimaging, immunological, and hormonal biomarkers^7^. It showed that only increased cortisol had a small predictive effect on recurrence, but even this effect disappeared when baseline clinical diagnoses, publication bias, or study quality were considered.

More recently, increasing focus has been on metabolic alterations in MDD^8^. Metabolomic and lipidomic studies showed more widespread alterations in MDD^9–11^, that have been hypothesized to constitute a trait associated with recurrence^12^. Recently, a targeted metabolomic study focusing on neurotransmitters and their metabolites in plasma, found a biochemical signature that could diagnose MDD-patients with up to 95% accuracy^13,14^. Moreover, metabolomics may predict MDD-recovery^15^, and response to therapy^16,17^. However, similar studies of biochemical signatures of remitted rMDD (rrMDD), with prospective follow-up to identify subjects at increased risk of future recurrence have not yet been conducted. The emerging recognition that the brain controls metabolism through neuroendocrine, autonomic, immune, and microbiome circuits^18^, implies that peripheral blood metabolomics can provide a uniquely accessible set of biomarkers that are diagnostic of real-time changes in brain-body function. This has now been shown in studies of myalgic encephalomyelitis/chronic fatigue syndrome^19^, schizophrenia^20^, Gulf War Illness^21^, response to treatment in autism spectrum disorder^22^, and treatment-refractory MDD with suicidal ideation^23^.

### Aims of the study

In this discovery phase pilot study, we tested the utility of metabolomic analysis for two purposes: (1) as a diagnostic tool to distinguish patients with rrMDD from controls, and (2) as a prognostic tool to assess the future risk of recurrence in patients with rMDD in drug-free remission at the time of sample collection.

## Patients and methods

### Study design

A cross-sectional patient-control design was used to compare medication-free rrMDD patients with age- and sex-matched, never-depressed controls to identify a metabolic profile of rrMDD patients^4^. In addition, rrMDD patients were followed prospectively every 4 months for 2.5 years by measuring depressive symptoms. Occurrence and time to recurrence of a new major depressive episode were documented.

### Approvals, inclusion and exclusion criteria

We included subjects who experienced ≥2 previous MDD-episodes according to the structured clinical interview for Diagnostic and Statistical Manual of Mental Disorders, fourth edition (DSM-IV) diagnoses (SCID), but were in stable remission (≥8 weeks 17-item Hamilton Depression Rating Scale (HAM-D) ≤ 7 (the lowest qualifying score for remission^24^), and not currently in an MDD-episode (SCID). Participants were 35–65 years, to include a homogeneous age group and minimize the risk of later conversion to bipolar disorder. Second, we included never-depressed controls without personal psychiatric history by SCID-analysis, or first-degree familial psychiatric history, matched to the rrMDD subjects for age, sex, educational level, working class and ethnicity. Both groups were recruited using identical advertisements in freely available sources and from previous studies^4^. This study was approved by the accredited Academic Medical Centre (AMC) Medical Ethical Committee (METC), and conformed to the Declaration of Helsinki^25^. Eleven of 40 healthy control females and 7 out of 19 healthy control males were recruited at the University of California, San Diego (UCSD) under IRB-approved protocol #140072 with signed informed consent. We excluded subjects with current diagnoses of alcohol and/or drug dependence, psychotic or bipolar symptoms, predominant anxiety or severe personality disorder. Other exclusion criteria included standard MRI-exclusion criteria, history of severe head trauma or neurological disease, or severe general physical illness. All participants had to be without psychoactive medication for ≥4 weeks.

### Psychometrics

At baseline we administered the SCID and HAM-D to check inclusion criteria and residual depressive symptoms. Subsequently, we followed-up rrMDD subjects using the SCID for 2.5 years to prospectively assess time to recurrence with ≥5 depressive symptoms lasting at least 2 weeks according to the DSM-IV criteria. HAM-D evaluations were conducted upon enrollment of 41 of the 59 healthy controls. Healthy controls were not followed prospectively.

### Metabolomics

Targeted, broad-spectrum, metabolomic analysis of 612 intracellular and plasma metabolites was performed by LC-MS/MS as described^26^ with minor modifications. A total of 399 of the 612 targeted metabolites were measurable in plasma of both males and females. This platform broadly interrogates 63 biochemical pathways and permits analysis of many of the metabolites known to be core features of the cell danger and integrated stress response (CDR and ISR)^27–29^.

See Supplementary Methods for additional methods

## Results

### Study cohort

**Fig. 1:**
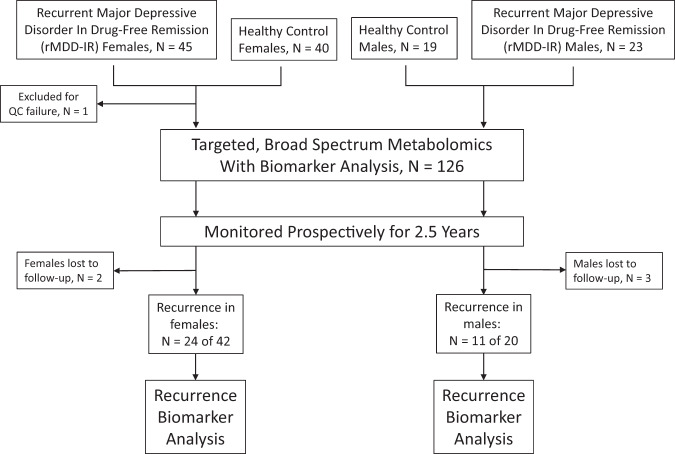
Flow chart of the study design. Metabolomic results from a total of 126 of 127 subjects enrolled were available for analysis. A total of 68 subjects with a history of recurrent major depressive disorder in remission (rrMDD) were followed prospectively for 2.5 years.

Sixty-eight drug-free rrMDD patients were enrolled and followed for 2.5 years (Flow diagram: Fig. 1). Fifty-nine age- and sex-matched controls were also enrolled.

### Participant characteristics

The rrMDD patients had a long history of illness (25 ± 2.4 years for males, and 27 ± 1.9 years for females), with a high total number of MDD-episodes (mean = 9.7 ± 2.5 for males; 8.1 ± 1.9 for females) (Table 1). Mean HAM-D score for the rrMDD subjects was 2 and 3 for males and females, respectively. A major epidemiologic difference between males and females with rrMDD was the median time to recurrence after enrollment (males: 5.5 months IQR = 1.9–7.6; females: 10.1 IQR = 4.9–17.2; Table 1). During follow-up three participants restarted antidepressants when experiencing a recurrence and two participants restarted antidepressants while in remission during follow-up. This occurred after blood sample collection for metabolomics at enrollment and did not affect the drug-free analysis.

**Table 1:** Participant characteristics.

	Males			Females			rrMDD
	rrMDD mean ± SEM (range)	CONTROLS mean ± SEM (range)	*p* value	rrMDD mean ± SEM (range)	CONTROLS mean ± SEM (range)	*p* value	Males vs. females *p* value
Subjects enrolled	23	19		45	40	–	–
Age	54 ± 1.4 (42–65)	56 ± 1.2 (46–64)	0.28	53 ± 1.2 (36–64)	51 ± 1.2 (36–65)	0.22	0.41
Metabolomic samples analyzed	23	19		44	40	–	–
Subjects living alone	9	5	0.51	19	13	0.38	0.99
Waist circumference (cm)	101 ± 2.4 (86–127)	104 ± 3.8 (91–129)	0.56	92 ± 2.1 (67–124)	85 ± 2.6 (57–111)	0.05*	0.01*
HAM-D score	2.0 ± 0.4 (0–6)	0.7 ± 0.4 (0–4)	0.02*	3.0 ± 0.3 (0–9)	1.2 ± 0.3 (0–5)	0.0003*	0.12
Age of 1st episode of MDD	30 ± 2.1 (9–43)	n/a		26 ± 1.7 (9–49)	n/a	–	0.15
Duration of rMDD (years)	25 ± 2.4 (6–50)	n/a		27 ± 1.9 (3–55)	n/a	–	0.39
Lifetime episodes of depression at entry	9.7 ± 2.5 (2–45)	n/a		8.1 ± 1.9 (2–60)	n/a	–	0.63
Years since last depressive episode	4.1 ± 0.8 (0.1–12)	n/a		5.1 ± 0.8 (0.1–21)	n/a	–	0.49
Subjects completing 2.5 years of follow-up	20	19	0.23	42	40	0.50	0.33
Subjects suffering recurrence	11	n/a		24	n/a		0.99
Median time to recurrence (IQR, months)^a^	5.5 (1.9–7.6)	n/a		10.1 (4.9–17.2)	n/a		0.02*
Subjects taking CNS medications^b^	1	0	0.99	3	1	0.62	0.99
Vitamins, supplements, and OTC medications	0.7 ± 0.19 (0–3)	0.6 ± 0.22 (0–2)	0.81	1.5 ± 0.22 (0–5)	0.9 ± 0.24 (0–5)	0.05*	0.03*
Non-CNS prescription medications	1.4 ± 0.36 (0–6)	0.5 ± 0.23 (0–2)	0.12	1.1 ± 0.24 (0–7)	0.3 ± 0.1 (0–2)	0.03*	0.46
Years of education^c^	16 ± 0.8 (9–20)	16 ± 1.2 (9–25)	0.90	14 ± 0.6 (9–20)	14 ± 0.6 (10–25)	0.22	0.04*
Ethnicity							
Northern European	22	19	0.99	35	34	0.42	0.08
Non-N. European	1	1	0.99	10	6	0.42	0.08

*rMDD* recurrent major depressive disorder in remission, *HAM-D* Hamilton depression rating scale, *OTC* over the counter, *CNS* central nervous system.

*Significant p value ≤ 0.05.

^a^Data from *N* = 24 females and 11 males with recurrence in 2.5 years post-enrollment. Excludes data from *N* = 18 females and 9 males without recurrence. ^b^No subjects were taking antidepressants (selective or non-selective serotonin or norepinephrine reuptake inhibitors, or tricyclics). The numbers indicate the 1–3 subjects taking low-dose benzodiazepines. These subjects stopped the medication for 1–2 days before the blood draw, depending on the half-life of the drug, then to restarted as indicated. ^c^Years of education: US system, High School=12; AA = 14; Bachelor’s=16; Master’s=18; JD = 19; MD, DO, or ND = 20; PhD=21; MD-PhD=25. Dutch system by Verhage score: 1 = 5 years, 2 = 6 years, 3 = 8 years, 4 = 9 years, 5 = 10 years, 6 = 15 years, 7 = 20 years.

### Metabolomics overview

**Fig. 2:**
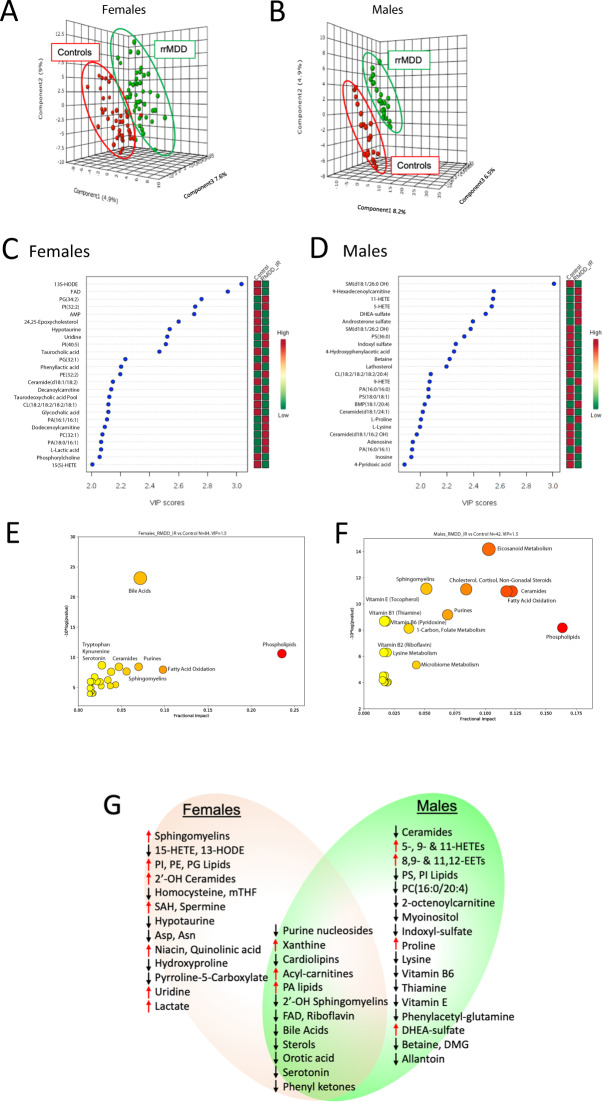
Metabolite and biochemical pathway abnormalities in recurrent major depressive disorder in remission. Females**: A**, **C**, **E**. Males**: B**, **D**, **F. AB**. Multivariate metabolomic discrimination of remitted recurrent major depressive disorder (rrMDD) from controls by partial least squares discriminant analysis. **CD**. Rank order of top 25 discriminating metabolites by variable importance in projection (VIP) scores. **EF**. Bubble impact plot of pathway alterations. **G**. Venn diagram of shared and gender-specific metabolites diagnostic for rrMDD. Red arrows indicate an increased, and black arrows indicate a decreased concentration was associated with rrMDD risk. VIP scores ≥1.5 were significant. rrMDD subjects *n* = 44 females, 23 males. Controls *n* = 40 females, 19 males.

Drug-free rrMDD subjects had a metabolic profile that could be distinguished from healthy controls. Multivariate analysis showed a clear separation between the 2 groups in both males and females (Fig. 2A, B). Top discriminating metabolites are shown in Fig. 2C, D. Relative metabolic impact and significance of these differences are shown in Fig. 2E, F. Tables S1 and S2 report the raw data and Tables S3 and S4 list the rank order of biochemical pathways that were disturbed in rrMDD female and male subjects, respectively, when compared to healthy age- and sex-matched controls. Figure 2G summarizes shared and gender-specific metabolic differences. Principal components analysis (PCA) showed that metabolomics explained up to 39.1% of the phenotypic variance between patients with rrMDD and healthy controls in both males and females (Fig. S1AC). Metabolomics explained up to 43.7% of the phenotypic variance between females who experienced recurrence of depressive symptoms and those with non-recurrence over the 2.5 years of prospective observation (Fig. S1B), and up to 50.6% in males (Fig. S1D).

Overall, alterations in lipid metabolism dominated the rrMDD metabolic signature. Lipid abnormalities constituted 80% of the top 10 pathway alterations in females (Table S3 and Fig. S1). In males, lipid abnormalities constituted 70% of the top 10 pathways (Table S4 and Fig. S2). The 8 lipid pathways most affected were phospholipids, fatty acids and acyl-carnitines, cardiolipins, two classes of sphingolipids (ceramides and sphingomyelins), eicosanoids, bile acids, sterols, and non-gonadal steroids.

The top non-lipid pathway alteration was purine metabolism (Tables S3 and S4 and Fig. S2). Pyrimidines, microbiome metabolites, GABA-glutamate-pyrroline-5-carboxylate-proline, folate-1-carbon, inositol, and tryptophan-serotonin metabolism were also altered in rrMDD subjects (Fig. 2 and S2). Gender-specific differences are discussed in Supplementary Results.

### Metabolic alterations shared by males and females

When analyzed at the pathway level,16 pathways were shared by both males and females with rrMDD (Fig. 2E, F and Tables S3 and S4). All 8 lipid pathways found to be abnormal were shared by both males and females. The other 8 shared pathways involved diverse non-lipid pathways.

#### Acyl-carnitines, cardiolipins, and vitamin B2

One of the most consistent lipid abnormalities found in both males and females was an increase in acyl-carnitines (Tables S3 and S4), which are a marker of decreased mitochondrial fatty acid oxidation^30^. In females, this was confined to increases in medium chain (C6–C10) acyl-carnitines, while in males, the long-chain (C12–C18), and very long-chain (≥C20) acyl-carnitines were increased (Tables S5 and S6). Cardiolipins, markers for inner mitochondrial membrane complexity and biomass, were also decreased. Phosphatidic acids (PA-lipids), precursors for cardiolipin synthesis, were increased in both males and females (Fig. 2G). Vitamin B2-metabolism needed for mitochondrial fatty acid oxidation was decreased in both males and females. In males this was reflected in a decrease of plasma riboflavin. In females, this was associated with a decrease in plasma flavin adenine dinucleotide (FAD; Fig. 2G, Tables S5 and S6).

#### Sphingolipids

Sphingolipids (ceramides, sphingomyelins, and glycosphingolipids) are major structural and signaling lipids that facilitate the exchange of materials between lysosomes and the plasma membrane to regulate cell growth and inflammation^31,32^, form membrane lipid rafts^33^, exosomes released from cells^34^, and are involved in synapses^35–37^. rrMDD males and females shared a specific abnormality in sphingolipid metabolism: a decrease in 2′-hydroxy sphingomyelin SM(d18:1/26:0 OH) (Fig. 2G). The 2′-hydroxylation (2′-OH) of the fatty acid precursor of the amide acyl chain of sphingolipids is catalyzed by the peroxisomal enzyme fatty acid 2′-hydroxylase (FA2H)^38^. 2′-hydroxy sphingomyelins are precursors for the 2′-OH glycosphingolipids needed for cell differentiation, neuronal connectivity, myelin stability, and have antitumor properties^39^.

#### Eicosanoids and oxylipins

Both males and females had alterations in eicosanoid (20-carbon, polyunsaturated) lipids made from arachidonic acid^40^, but the specific metabolites and the direction of change differed. Females also showed a decrease in an 18-carbon oxylipin made from linoleic acid^41^ called 13(S)-hydroxyoctadecadienoic acid (13-HODE), which was unchanged in males. 15(S)-hydroxyeicosatetraenoic acid (15(S)-HETE) was decreased in females, but not in males (Fig. 2C and Tables S5 and S6). 13-HODE and 15-HETE are anti-inflammatory and pro-resolving oxylipins that also have antitumor effects^42^. In contrast, rrMMD-males had increases in 3 eicosanoids: 11-HETE, 9-HETE, and 5-HETE, which are pro-inflammatory mediators made by neutrophils, eosinophils, and mast cells^43,44^. Males also had an increase in the vasodilatory and anti-inflammatory epoxyeicosatrienoic acids 8,9-EET, and 11,12-EET (Fig. 2G). The large number of alterations in eicosanoid metabolism made this the most statistically significant pathway alteration in males (Fig. 2F).

#### Sterols

Sterols are needed for the synthesis of cholesterol, glucocorticoid, and steroid hormones and bile acids. Sterol synthesis requires coordinated enzyme activity in the endoplasmic reticulum (ER) and mitochondria. Sterols were decreased in both male and female rrMDD subjects (Fig. 2G, Tables S3 and S4). In females, both 24,25-epoxycholesterol and cholesteryl-sulfate were decreased. In males, cholesterol precursors 24,25-dihydrolanosterol and lathosterol were decreased (Tables S5 and S6).

#### Bile acids

Bile acid metabolism requires coordinated activities of enzymes located in mitochondria, peroxisomes, ER, and the gut microbiome^45^. Bile acids are signaling molecules that bind to several classes of nuclear receptors (FXR, PXR, and CAR), and permit real-time coordination between food intake, the microbiome, liver, and systemic detoxification systems^46^. Bile acids are made from cholesterol and represent the major disposal route for excess cholesterol. Both males and females had decreased plasma levels of bile acids. rrMDD-females had decreased levels of four glycine- and taurine-conjugated secondary bile acids including glycocholic and taurocholic acids (Tables S3–S6 and Fig. 2). Bile acid abnormalities were the most statistically significant single pathway alteration in females (Fig. 2E). rrMDD males had decreased levels of the secondary bile acid, deoxycholic acid, which is formed by dehydroxylation of cholic acid by normal gut bacteria.

#### Purines

Purine nucleosides adenosine, guanosine, and inosine were decreased and xanthine was increased in both males and females with rrMDD (Fig. 2G and Tables S3 and S4). Plasma purine nucleosides are derived by dephosphorylation of purine nucleotides like ATP, ADP, AMP, GMP, and IMP. Xanthine is a purine nucleobase that has been shown to connect purine metabolism with the immune system, memory, and anxiety^47^. De novo synthesis, salvage, and metabolism of purine nucleotides depends on cooperative activities of extracellular, cell membrane-associated, cytosolic, and mitochondrial enzymes.

### Metabolomics as a diagnostic tool

Area under the receiver operator curve (AUROC)-analysis was used to test the accuracy of metabolites to distinguish between rrMDD subjects and healthy controls (Fig. S3). The classifier for females used 12 metabolites, resulting in an AUC of 0.83 (95%Cl = 0.68–0.96; sensitivity = 80%, 95%Cl = 0.66–0.89; specificity = 87%, 95%Cl = 0.74–0.95). For males, seven metabolites were used as classifier, resulting in an AUC of 0.83 (95%Cl = 0.64–1.0; sensitivity = 74%, 95%Cl = 0.53–0.87; specificity = 79%, 95%Cl = 0.57–0.91).

### Metabolomics as a prognostic tool

#### Predictors shared by males and females

**Fig. 3:**
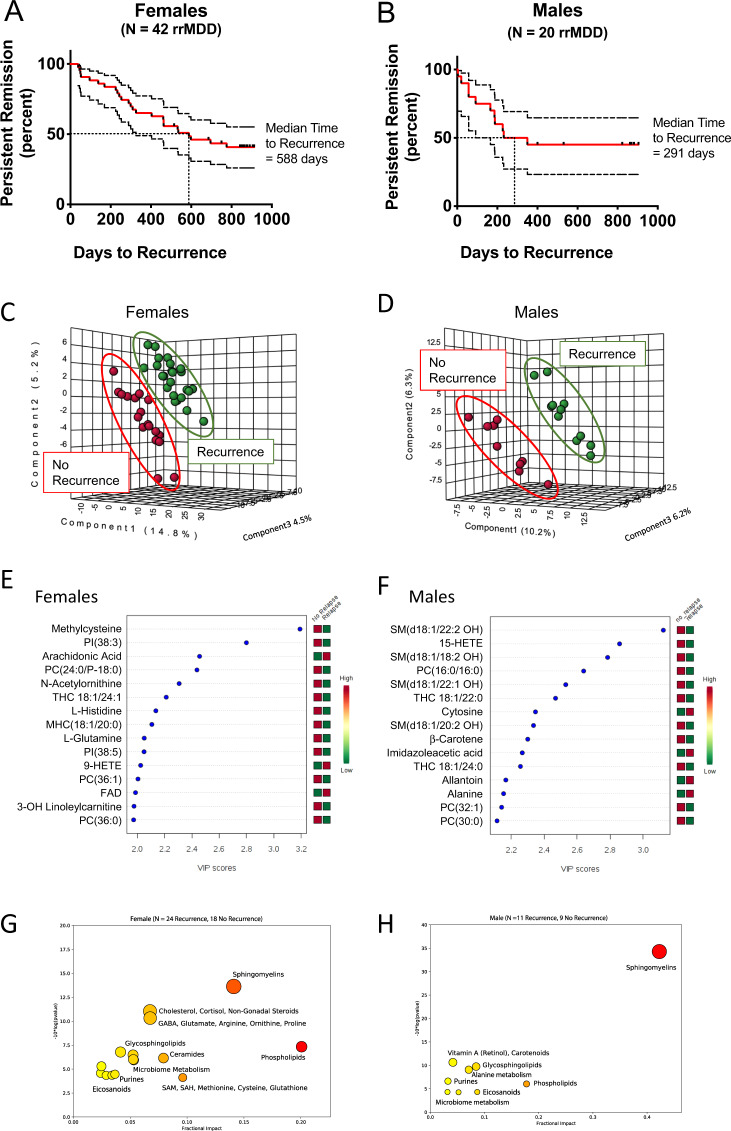

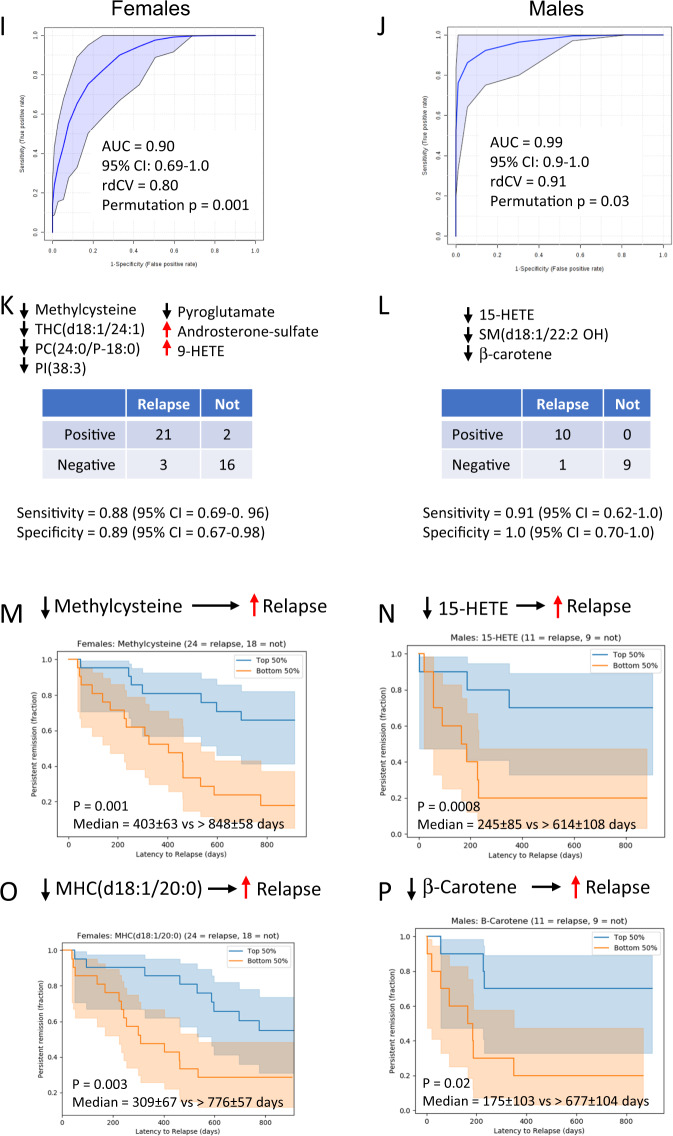
Metabolomic predictors of recurrence in remitted recurrent major depressive disorder (rrMDD). **A** Kaplan–Meier analysis of latency to recurrence in subjects with remitted recurrent major depressive disorder (rrMDD), Females, **B** Males. Dotted boundaries indicate the 95% confidence intervals. Metabolic predictors of recurrence in recurrent major depressive disorder. Females: **C**, **E**, **G**. Males: **D**, **F**, **H**. **CD** Multivariate metabolomic discrimination of subjects with rMDD who experienced recurrence in the next 2.5 years, and those who did not, analyzed by partial least squares discriminant analysis. **EF** Rank order of top 15 discriminating metabolites by variable importance in projection (VIP) scores. **GH** Bubble impact plot of pathway alterations. Receiver operator characteristic (ROC) curve analysis of multianalyte diagnostic classifiers for rrMDD. **I**. The classifier for females used 7 metabolites. **J** The classifier for males used three metabolites. AUC area under the curve, rdCV repeated double cross validation accuracy. **KL** 2 × 2 contingency table analysis. Cox proportional hazard analysis of selected metabolites. **M** Decreased methylcysteine predicted a higher risk of recurrence in females. **N** Decreased 15-hydroxyeicosatetraenoic acid (15-HETE) predicted a higher risk of recurrence in males. **O** Decreased monohexosyl ceramide (MHC(d18:1/20:0)) predicted a higher risk of recurrence in females. **P** Decreased β-carotene predicted a higher risk of recurrence in males. rrMDD subjects were followed prospectively for 2.5 years: *n* = 42 females (24 with recurrence, 18 no recurrence), 20 males (11 with recurrence, 9 no recurrence).

Median time to recurrence during the 2.5 years follow-up for all rrMDD subjects was 588 days for females and 291 days for males (Kaplan–Meier analyses; Fig. 3A, B). Multivariate analysis permitted the metabolomic signature of subjects who experienced recurrence to be distinguished from those who did not (Fig. 3C, D and Tables S7 and S8). The top discriminating metabolites are shown in Fig. 3E, F. Overall, most predictive metabolite classes for both genders were sphingomyelins and phospholipids (Fig. 3G, H). AUROC-analysis was used to test the prognostic accuracy of a female-specific classifier using 7 metabolites and a male-specific classifier that used 3 metabolites (Fig. 3I, J). The predictive accuracy for recurrence in females was 0.90 (95%CI = 0.69–1.0; sensitivity = 0.88; specificity = 0.89; Fig. 3I, K). Although different from female rrMDD subjects, the predictive accuracy for recurrence of depression in males was 0.99 (95% CI = 0.9–1.0; sensitivity = 0.91; specificity = 1.0; Fig. 3J, L).

**Fig. 4:**
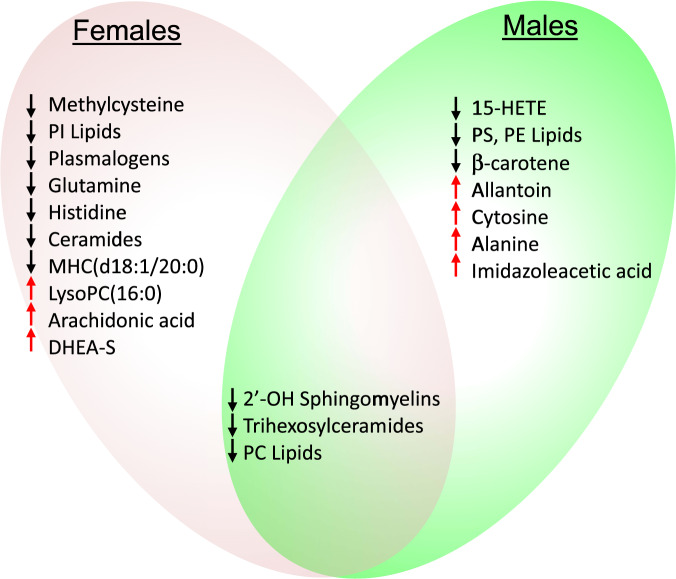
Venn diagram of shared and gender-specific predictors of recurrence. Red arrows indicate an increased, and black arrows indicate a decreased concentration was associated with risk of recurrence of depression. rrMDD subjects were followed prospectively for 2.5 years: *n* = 42 females (24 with recurrence, 18 no recurrence), 20 males (11 with recurrence, 9 no recurrence).

Three metabolite classes predicted recurrence risk in both males and females by Cox proportional hazard analysis (Fig. 4 and Tables S7–S10). These were a decrease in 2′-hydroxy sphingomyelins (2′-OH SM), trihexosylceramides (THC), and phosphatidylcholine (PC) lipids. In both males and females, nearly 80% of the predictive metabolites identified had a negative correlation with recurrence risk (Tables S9 and S10). This means that when the blood level was low compared to the other rrMDD subjects, recurrence risk was higher, and conversely.

#### Female-specific predictors of recurrence

All of the strongest predictors of recurrence were gender specific. In females the strongest predictor was low methylcysteine (Fig. 3K, M and Table S9). Female rrMDD subjects in the bottom half of plasma methylcysteine were found to have a median time to recurrence of 1.1 years (403 ± 63 days, mean ± SEM). Women in the top 50th methylcysteine percentile were much slower to experience recurrence (median = 2.3 years; 848 ± 58 days; Cox beta coefficient = −1.8; *p* < 0.00006; Table S9 and Fig. 3M). Monohexosylceramide (MHC(d18:1/20:0)) was another example of a metabolite that protected against future recurrence (Fig. 3O and Table S9). Two metabolites that increased recurrence risk were lysophosphatidylcholine 16:0 (LysoPC(16:0)) and arachidonic acid (20:4). These two lipids can be produced from the same parental phosphatidylcholine lipid, PC(16:0/20:4) through the action of lipoprotein associated phospholipase A2 (LP-PLA_2_) and other PLA_2_-types under conditions of stress^48^. Metabolites found to increase and decrease recurrence risk are listed in Table S9. Of the 399 measured metabolites, 33 were significant predictors of recurrence risk in females, and in 82% of these metabolites (27/33) higher levels decreased recurrence risk (Table S7 and S9). Correcting for number of previous episodes and residual symptoms did not change hazard-ratios of these metabolites.

#### Male-specific predictors of recurrence

Of the 399 measured metabolites, 17 were significant predictors of recurrence risk in males (Table S8 and S10). The eicosanoid lipid, 15-hydroxyeicosatetraenoic acid (15-HETE) was the top predictor of recurrence in males. When males were stratified into top and bottom 50th percentiles, rrMDD subjects in the bottom half for this plasma 15-HETE were found to have a median time to recurrence of 0.7 years (245 ± 85 days). Males in the top 50th percentile for this anti-inflammatory eicosanoid were much slower to experience recurrence (median = 1.7 years; 614 ± 108 days; Fig. 3N; Cox beta coefficient = −1.5; *p* < 0.0006; Table S10). Beta carotene was also protective. Males in the lowest 50th percentile for plasma beta-carotene had a median time to recurrence of 0.5 years (175 ± 103 days), while the upper 50th percentile had a median time to recurrence of 1.9 years (677 ± 104 days; Fig. 3N and P; Table S8 and S10). Increased alanine and allantoin were associated with an increased risk of recurrence in males. Increased alanine is produced as a transamination product of pyruvate and is a marker of mitochondrial dysfunction^49^. Allantoin is produced non-enzymatically from uric acid by exposure to reactive oxygen species (ROS) and is a marker of oxidative shielding^50^ and stress^51^. Increased levels of 14 of 18 (78%) predictors in males decreased recurrence risk. Figures S4 and S5 illustrate the Kaplan–Meier style recurrence profiles of the top predictive metabolites determined by Cox proportional hazard analysis in females and males, respectively. Correcting for number of previous episodes and residual symptoms did not change the hazard-ratios of these metabolites, except for beta-carotene, SM(d18:1/20:2 OH), PS(18:0/18:1), PE(34:1), or SM(d18:1/20:1), where correction for especially previous lifetime episodes of depression increased the hazard-ratios.

### Sample size calculation for validation studies

Three methods were used to estimate the sample size needed in future studies to validate the results of this pilot study (see Supplemental Methods). The median Pearson *r* for the metabolites in females having VIP scores ≥1.5 was *r* = 0.2. The median Z-score difference was 0.39 (Tables S5, S11–S14). Using correlation analysis and a requirement for a Pearson *r* ≥ 0.2, the total number of subjects of a single sex (cases plus controls) was 194. Using multiple regression analysis and a threshold of at least 35 significant metabolites and a Cohen’s *f*^*2*^ = 0.15, the study size was 201. Using a Z-score threshold of ≥0.4 (significant metabolites in cases must differ from controls by at least 0.4 standard deviations), the study size was 198. The mean estimate was 198 ± 3.5 (mean ± sd). These results showed that a validation-scale study of at least 198 females and 198 males (99 cases and 99 controls each) will be needed to confirm the results.

See Supplementary Results for additional results.

## Discussion

Metabolomic analysis revealed an underlying biochemical signature in remitted recurrent major depressive disorder (rrMDD) that distinguished patients from healthy controls. This difference was psychometrically inapparent as patients were studied during antidepressant-free remission. Patterns of metabolic abnormalities that we found reflected alterations in chemical communication across lipid membranes and between organelles, cells, organ systems, and the microbiome. Lipid abnormalities constituted 60–70% of the total metabolic impact. Even more striking was the finding that metabolomic analysis was able to unmask a latent signature of future risk of recurrence with 90–99% accuracy. If replicated, this finding could have a significant impact on clinical practice by permitting patients with major depressive disorder to be stratified according to future risk, and new preventive treatments to be tested systematically in clinical trials. To the best of our knowledge, this is the first report to show that broad-spectrum targeted metabolomics can predict future recurrence risk.

Interestingly, the top metabolites that distinguished rrMDD cross-sectionally from controls were not the same biomarkers that predicted future recurrence risk. Reciprocally, the top predictors of future risk were not the same biomarkers that distinguished patients with recurrent MDD in drug-free remission cross-sectionally from healthy controls. A similar pattern of metabolites being used differently in health than in disease has been observed previously for other biological markers. For example, a study showed that cortisol^52^ was increased in rrMDD subjects compared to controls, while decreased cortisol predicted recurrence in the same study. In this example, higher levels of cortisol actually protected against future recurrence in patients at risk. Altogether, these differences between diagnostic and prognostic biomarkers makes it unlikely that these observations merely represent epiphenomena or consequences of earlier MDD episodes.

### Measuring latent risk

These data suggest that once a new disease state such as MDD has been entered, asymptomatic remission comes with a latent (hidden) future risk that can be objectively measured, permitting patients to be stratified. In this new state of latent risk, biochemical and endocrine pathways are used differently to prevent disease progression and recurrence than in healthy control subjects. These results suggest that natural recovery from disease is not the simple reversal of the sequence of pathogenic events that led to disease. Metabolomic analysis unmasked new interaction patterns between metabolites that changed the future risk in patients in a disease state, but that had either no utility, or even an opposite effect, in predicting the future risk of developing that disease state in healthy controls^53^. Rephrasing this, the clinical context of the patient in health or disease determines the meaning of the data, and the pathophysiologic sequence of events that produced the original disease is different than the path that is used to minimize future complications, or to recover and heal from that disease^18^.

### Mechanistic implications

Membrane properties are determined by lipids and transporters that conduct material across those membranes. Changes in membrane-dependent cell-to-cell and interorganellar communication provide a rationale for why over half of all the prognostic metabolites for recurrence risk in rrMDD were lipids, and why those that were not lipids, like purines, are potent regulators of membrane and transporter properties. We found that several nucleosides of purines such as inosine and guanosine were decreased while certain nucleobases like xanthine were increased. This has been reported in independent studies of MDD^54^ and confirms an important role of purines and purinergic signaling in regulating affective and several other neuropsychiatric and neurodegenerative disorders^55,56^. Metabolic abnormalities found in rrMDD support the notion that interorganellar and intercellular exchange and the transformation of metabolites across membranes—the connectivity and communication between organelles, cells, and organ systems—is altered. Impaired communication across membranes, as measured by plasma metabolomics, permits the pathogenesis of major depressive disorder to be reframed as a neurometabolic disorder^8,57,58^. When reframed under this new paradigm as a neurometabolic disorder, novel approaches to treatment become apparent that may never have been considered under the old paradigm of MDD as an isolated disorder of brain function independent of whole-body changes in metabolism.

### The mitochondrial nexus

Mitochondria have been shown to coordinate many of the metabolic features of stress that can play a causal role in mental health disorders^27,59,60^. Mitochondria are the hub of the wheel of cellular metabolism. This nexus point is a crossroads for many neurodevelopmental and psychiatric disorders^61^. Mitochondria naturally respond to chemical and physical environmental changes, making their membrane structures of fundamental interest^62^. Although typically thought of as an energy factory, mitochondria catalyze over 700 biochemical reactions^63^ needed to produce building blocks for cell growth, repair, and signaling. Mitochondria can also shift dynamically between three major modes of function, or functional types designated M1-, M0-, and M2-organelles, according to cellular needs^18^. Cells in which M1- and M0-mitochondria predominate are pro-inflammatory and dependent on glycolytic metabolism for energy. M1- and M0-cells release more lactic acid in order to maintain intracellular redox. Lactic acid elevation was found in females with a history of rMDD in this study (Fig. 2G), and is a known feature of symptomatic depression that decreases with treatment^64,65^. Moreover, both males and females had several other markers of decreased mitochondrial function and/or biomass. These included decreased purines like adenosine, inosine, and guanosine, and decreases in the pyrimidine precursor orotic acid, and decreased cardiolipins, which are markers of inner mitochondrial membrane surface area and biomass. Mitochondria specialized for oxidative phosphorylation are designated M2-organelles, needed for burning fatty acids for energy to make ATP. When M2-mitochondrial function is impaired, several fatty acyl-carnitines increase in the blood^30^. Acyl-carnitines were increased in both males and females with antidepressant-free rrMDD, and the major vitamins needed for fatty acid oxidation (riboflavin and FAD) were decreased. Disturbances in mitochondrial function leading to acyl-carnitine abnormalities have been observed in several other independent studies of MDD^66^. These findings support the hypothesis that even in remitted rMDD a shift in mitochondrial function exists and persists. This change is functionally dynamic, shifting mitochondria away from the M2-polarized oxidative phosphorylation phenotype, to the M1 and M0 phenotypes associated with inflammation and proliferation, respectively^18,63^.

### Study limitations

A limitation of this study was the relatively smaller number of rrMDD subjects enrolled. This was a greater problem for males than for females. The smaller sample size for males captured a smaller proportion of the natural phenotypic variation, making the results vulnerable to overfitting. The small sample size influenced the calculated false discovery rates (FDRs). FDRs for the significant metabolites in females ranged from 0.6 to 0.96. FDRs for the 54 discriminating metabolites in males ranged from 0.82 to 0.91. As expected for a small-scale pilot study, these FDRs were higher than would be desired for a validation study. To further test the statistical validity of these results we used repeated double cross validation (rdCV) analysis. rdCV analysis showed modest values of 0.74–0.76 for males and females in remission, while a similar analysis of the prognostic classifiers for high and low risk of future recurrence of MDD symptoms reached rdCV values of 0.91 and 0.80 for males and females, respectively. Despite these high values for the in-sample validation statistics, the generalizability of these findings is not currently known, and will require independent testing in future cohorts. Larger studies will be needed for validation, and verification of the sensitivity and specificity of our results. Moreover, we did not include first-episode MDD-patients. Although this allowed focus on recurrent patients with a high recurrence risk, it precluded comparative analyses with patients with a relatively low risk. Future studies applying comprehensive metabolomics in untreated first-episode MDD may further elucidate the role of different metabolites in the prognosis of MDD.

See Supplementary Discussion for additional discussion

## Conclusions

Targeted broad-spectrum LC-MS/MS-metabolomics of 399 plasma metabolites was used to study recurrent major depressive disorder in remission (rrMDD), and the time to recurrence, in 126 cases and controls. Lipid abnormalities dominated rrMDD’s metabolic signature. The most powerful statistical inferences came from the 2.5-year prospective study of subjects at risk for recurrence. Stratification of rrMDD subjects using metabolomics was able to predict recurrence risk with >90% accuracy in both males and females. Nearly 80% of the metabolites with greatest predictive accuracy for recurrence risk belonged to just six pathways: phospholipids, sphingomyelins, glycosphingolipids, eicosanoids, microbiome, and purines. Abnormalities in these pathways support the emerging conceptual framework that mitochondria act as a metabolic nexus^61^—as sensors and regulators of cell function that respond to environmental threat, stress, or injury^18,27,62^. After correcting for residual symptoms and lifetime MDD-episodes, metabolomic analysis was found to add new information that was not statistically correlated with any other neuropsychiatric parameter measured during remission and was highly significant in prospectively identifying the rrMDD patients at greatest risk of recurrence. New clinical trials designed to restore normal lipid metabolism^67^, mitochondrial health^68^, metabokine signaling^18,22^, and microbiome health^69,70^ through dietary, supplement, psychological stress reduction, and medical interventions will be required to confirm these results.

## Supplementary information

Supplementary Information

Supplementary Tables S1-S14

## Data Availability

Raw AUC data from the LC-MS/MS analysis and recurrence data are provided in Tables S1 and S2.
