# Correction to: Metabolic features of recurrent major depressive disorder in remission, and the risk of future recurrence

**DOI:** 10.1038/s41398-021-01239-4

**Published:** 2021-02-08

**Authors:** Roel J. T. Mocking, Jane C. Naviaux, Kefeng Li, Lin Wang, Jonathan M. Monk, A. Taylor Bright, Caroline A. Figueroa, Aart H. Schene, Henricus G. Ruhé, Johanna Assies, Robert K. Naviaux

**Affiliations:** 1 Department of Psychiatry, Amsterdam UMC, Academic Medical Center, University of Amsterdam, Meibergdreef 5, 1105 AZ Amsterdam, The Netherlands; 2 The Mitochondrial and Metabolic Disease Center, University of California, San Diego School of Medicine, 214 Dickinson St., Bldg CTF, Rm C107, San Diego, CA 92103-8467 USA; 3 Department of Neurosciences, University of California, San Diego School of Medicine, 214 Dickinson St., Bldg CTF, Rm C107, San Diego, CA 92103-8467 USA; 4 Department of Medicine, University of California, San Diego School of Medicine, 214 Dickinson St., Bldg CTF, Rm C107, San Diego, CA 92103-8467 USA; 5 Department of Psychiatry, Radboud University Medical Center, Nijmegen, the Netherlands; 6 Donders Institute for Brain, Cognition and Behavior, Radboud University Nijmegen, Nijmegen, the Netherlands; 7 Department of Pediatrics, University of California, San Diego School of Medicine, 214 Dickinson St., Bldg CTF, Rm C107, San Diego, CA 92103-8467 USA; 8 Department of Pathology, University of California, San Diego School of Medicine, 214 Dickinson St., Bldg CTF, Rm C107, San Diego, CA 92103-8467 USA; 9 Present Address: Colt Neck Labs, 838 E High St 202., Lexington, KY 40503 USA; 10 Present Address: School of Social Welfare, University of California, Berkeley, CA 94720 USA

**Keywords:** Predictive markers, Depression

Correction to: *Translational Psychiatry*

10.1038/s41398-020-01182-w published online 11 Jan 2021

The original version of this article unfortunately contained a mistake. The pdf version of the article was missing the correct Fig. 4. Instead, the pdf used the Fig. 4 title and legend on page 10 for Fig. 3 panels I–P. The Venn diagram in the true Fig. 4 has been dropped from the pdf. The correct figure can be found below. We apologize for the mistake. The original article has been corrected.

**Fig. 4:**
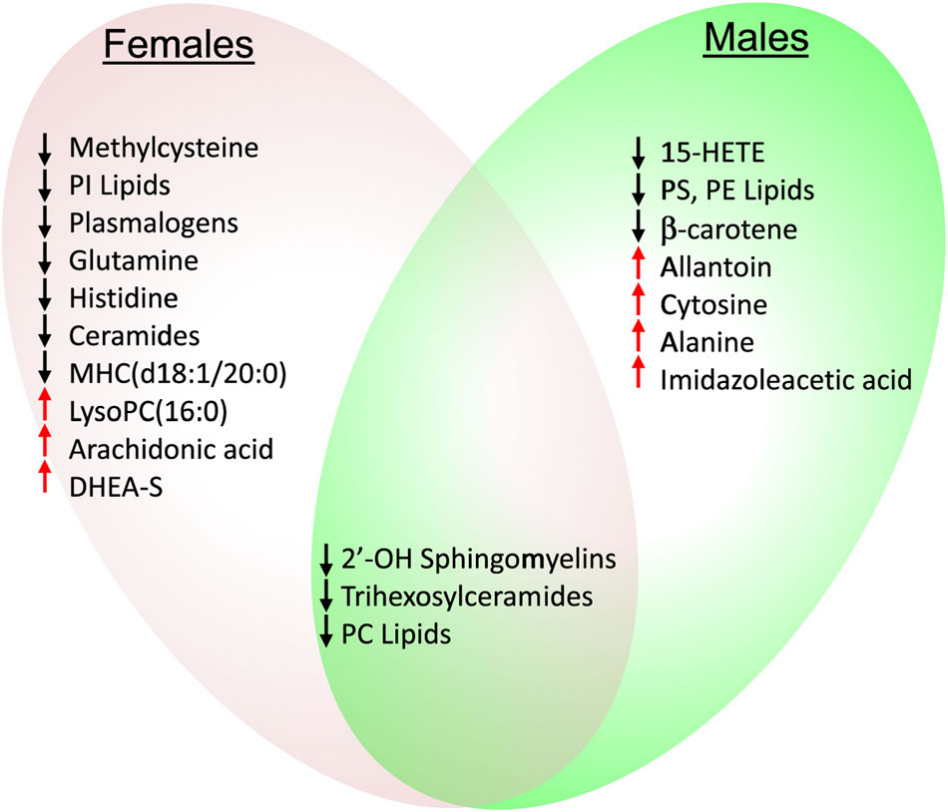
Venn diagram of shared and gender-specific predictors of recurrence. Red arrows indicate an increased, and black arrows indicate a decreased concentration was associated with risk of recurrence of depression. rrMDD subjects were followed prospectively for 2.5 years: *n* = 42 females (24 with recurrence, 18 no recurrence), 20 males (11 with recurrence, 9 no recurrence).

