# Supplementary material for: Metabolic features of recurrent major depressive disorder in remission, and the risk of future recurrence
Source: Transl Psychiatry. 2021 Jan 11;11:37. doi: 10.1038/s41398-020-01182-w (PMC7801396; doi:10.1038/s41398-020-01182-w)
Supplement: Supplementary file 1 — Supplementary Information [file 41398_2020_1182_MOESM1_ESM.pdf]

Supplementary Information
Translational Psychiatry
10 December 2020

Metabolic Features of Recurrent Major Depressive Disorder in Remission, and the Risk of Future Relapse

Roel J. T. Mocking; Jane C. Naviaux; Kefeng Li; Lin Wang; Jonathan Monk; A. Taylor Bright; Caroline A. Figueroa; Aart H. Schene; Henricus G. Ruhé; Johanna Assies; Robert K. Naviaux

SUPPLEMENTARY METHODS

Study Enrollment

Participants were enrolled between December 1, 2011 and November 20, 2014. Plasma samples were analyzed in September 2015.

Sample Collection

Venous blood was collected at baseline between the hours of 8 am and 1 pm, after an overnight fast, into lithium-heparin vacutainer tubes. Plasma was separated by centrifugation at 1500g for 10 minutes at room temperature within one hour of collection, aliquoted into 0.5 ml samples and stored at -80˚C in cryotubes until analysis.

Statistical Analysis

Demographic data were analyzed by t-tests or non-parametric Mann-Whitney U tests. Categorical data was analyzed by Fisher's exact test. Peak area under the curve (AUC) data from metabolomics were $\log_2$ transformed, scaled by control standard deviations, and the resulting z-scores (Tables S11-S14) analyzed by multivariate partial least squares discriminant analysis (PLSDA) in MetaboAnalyst [1,2]. Post hoc correction for multiple hypothesis testing was done by the false discovery rate (FDR)

method of Benjamini and Hochberg [3]. Bayesian false discovery rates were estimated using the Storey q value [4]. Metabolites with PLSDA variable importance in projection (VIP) scores ≥ 1.5 were considered significant. Significant metabolites were grouped into pathways and their VIP scores summed to determine the rank-ordered significance of each biochemical pathway. Random forest analysis [5] was used to rank metabolites for their ability to distinguish rrMDD cases and controls using mean decrease in accuracy (MDA) scores. *k*-nearest neighbor (*k*-NN) clustering was used to identify metabolite groups that contributed in different ways to the discrimination of rrMDD and controls. Time to recurrence was evaluated by Kaplan-Meier analysis. Models for future risk stratification were constructed using Cox proportional hazard methods [6]. Significant prognostic metabolites were identified by Cox analysis with p values < 0.05, were aggregated into biochemical pathways, and ranked according to the sum of the absolute value of the Cox β coefficients as a measure of predictive impact. Hazard ratios were corrected for residual symptoms using the HAM-D scores and the natural log of the lifetime episodes of depression as previously described [7]. Classifiers of 5-15 metabolites were selected and tested for diagnostic accuracy using area under the receiver operator characteristic (AUROC) curve and random forest analysis. Confidence intervals for the ROC curves were calculated by bootstrap resampling. Sample size calculations for future validation studies were performed using three methods: 1) Correlation analysis; two-sided $\alpha = 0.05$, $\beta = 0.2$ (power = 0.8), and a Pearson r ≥ 0.2 to calculate the number of cases and controls needed (*n* = 194; https://sample-size.net/all-calculators-on-this-site/), 2) Multiple regression analysis using two-sided $\alpha = 0.05$, power = 0.8, and F-test Cohen's $f^2 = 0.15$, to detect at least 35 significant metabolites (*n* = 201; https://www.danielsoper.com/statcalc/calculator.aspx?id=1), and 3) Z-score analysis; two-sided $\alpha = 0.05$, $\beta = 0.2$, and a metabolite z-score difference of ≥ 0.4 (*sd* = 1; *n* = 198; https://sample-size.net/sample-size-means/). Classifiers were validated within sample using repeated double cross validation (rdCV) [8], with bootstrapping 100 times to test random subsamples of 2/3 in and 1/3 out, and by permutation analysis [9]. Results were organized into biochemical pathways and visualized in Cytoscape version 3.4.0. Principal components analysis (PCA) and scree plots were used to quantify the fractional contribution of metabolomics to phenotypic variance. Statistical methods were

implemented in Stata (Stata/SE12.1, StataCorp, College Station, TX), Prism (Prism 6, GraphPad Software, La Jolla, CA), Python, or R.

SUPPLEMENTARY RESULTS

Gender-Specific Differences

Many of the strongest metabolic changes found in rrMDD were gender-specific (Figure 2G, S3, and S4). Gender-specific differences in the metabolic response in major depressive disorder have been documented in independent studies[10]. Ten of these are grouped by pathway and discussed below.

Phospholipids

Although 15 of the same biochemical pathways were disturbed in both males and females, the direction of change was sometimes different. All the major phospholipid classes found to be abnormal in females (phosphatidic acid (PA), phosphatidylglycerol (PG), phosphatidylinositol (PI), phosphatidylcholine (PC), and phosphatidylethanoloamine (PE)) were increased, except for cardiolipin which was decreased. Myoinositol, a precursor for PI lipids was not significantly decreased in females. The only phospholipid intermediate that was decreased in females was phosphorylcholine (Table S3 and S5). In contrast, PC(16:0/20:4), PS, and PI lipids, and myoinositol were decreased in males (Table S4 and S6).

Non-gonadal steroids

The sulfated forms of the non-gonadal, androgenic steroids androsterone-sulfate and dehydroepiandrosterone-sulfate (DHEA-S) were both increased in males with rrMDD, but not in females (Table S3-S6).

Eicosanoids and Oxylipins

Eicosanoids are a major class of pro-inflammatory and anti-inflammatory lipids synthesized from 20-carbon, polyunsaturated fatty acids, including the omega-6 fatty acid, arachidonic acid (C20:4), and 20-carbon omega-3 and omega-9 fatty acids. 13(S)-hydroxyoctadecadienoic acid (13-HODE) was decreased in females, but not changed in males. Similarly, 15-hydroxyeicosatetraenoic acid (15-HETE) was decreased in females, but not in males (Figure 2C-G, Table S3, S4). 13-HODE and 15-HETE are anti-inflammatory and pro-resolving oxylipins that also have antitumor effects [11]. In contrast, males with rrMDD had increases in 3 eicosanoids: 11-HETE, 9-HETE, and 5-HETE, which are proinflammatory mediators made by neutrophils, eosinophils, and mast cells [12,13]. Males also had an increase in the vasodilatory and anti-inflammatory epoxyeicosatrienoic acids 8,9-EET, and 11,12-EET, but females did not (Figure 2CD and G). The large number of disturbances in eicosanoid metabolism made this the most statistically significant single pathway disturbance in males (Figure 2F).

Acyl-carnitines and lactate

Associated with the decreased mitochondrial medium chain (C4-C10) fatty acid oxidation capacity in females was an increase in medium chain acyl-carnitines and L-carnitine. There was also an increase in lactate, reflecting an increase in glycolytic ATP production. While males had a defect in mitochondrial long-chain (C12-C18) fatty acid oxidation and an associated increase in long-chain acyl-carnitines. Lactate was not significantly increased in males ($p = 0.22$; Figure 2D, Table S4). A C8 medium chain, monounsaturated acyl-carnitine (2-octenoylcarnitine) known to be associated with obesity and risk of diabetes [14] was decreased in males but not in females with rrMDD (Figure 2G).

Sphingolipids

Sphingolipid metabolism, including ceramides and sphingomyelins, was disturbed in both males and females with a history of recurrent major depressive disorder. However, in females, the non-2’-hydroxylated sphingomyelins were increased, while the 2’-hydroxy sphingomyelins were decreased

(Table S3 and S5). In males, only the 2'-hydroxysphingomyelins were decreased and other sphingomyelins were unchanged (Figure 2G, Table S4 and S6).

Folate, 1-Carbon, methylation, transsulfuration, and polyamine metabolism

Although males and females with rrMDD shared disturbances in folate-1-carbon metabolism, the metabolites used were different. Females had decreases in 5-methyltetrahydrofolic acid (mTHF), homocysteine, and hypotaurine, with an increase in the methylation inhibitor S-adenosylhomocysteine (SAH). The polyamine spermine was also increased in females but not males (Figure 2G, Table S3-S6). Spermine synthesis depends on the decarboxylation of S-adenosylmethionine (SAM) to dcSAM and its use as a 3-carbon aminopropyl donor in polyamine synthesis.

Tryptophan and indole metabolism

In addition to a decrease in plasma serotonin, females with rrMDD also had an increase in the pro-inflammatory and neurotoxic metabolite of tryptophan, quinolinic acid and its downstream product, the vitamin B3 redox cofactor, niacinamide (Figure 2G, Tables S3-S6). Males did not. Indoxyl-sulfate, a metabolite regulated by both microbiome and liver metabolism, was decreased in males but not in females (Figure 2G).

Asparagine and aspartate metabolism

Both asparagine, and it biosynthetic precursor aspartate, were decreased in females with rrMDD, but were unchanged in males (Figure 2G, Tables S3-S6).

Collagen, hydroxyproline, lysine, proline, and pyrroline-5-carboxylate

Hydroxyproline is a marker of collagen turnover and vitamin C metabolism and was decreased in females with rrMDD, but unchanged in males (Figure 2G, Tables S3 and S4). In males, two other amino acids that are enriched in collagen, proline and lysine, were dysregulated in opposing directions. Lysine was decreased and proline was increased in males but were unchanged in females.

In females, the stress-associated oxidation product of proline, pyrroline-5-carboxylate (P5C), was decreased. P5C was not changed in males (Figure 2G, Tables S3-S6).

Vitamin metabolism (B1, B6, E)

Vitamins B1 (thiamine), a vitamin B6 metabolite (4-pyridoxic acid), and the trimethylated α-tocopherol, a readily absorbed form of vitamin E, were decreased in males, but were unchanged in females with rrMDD (Figure 2G, Tables S3-S6).

Metabolic Alterations Shared by Males and Females

Pyrimidines

Orotic acid was decreased in both males and females with rrMDD (Figure 2G). Orotic acid is the product of the 4th step of *de novo* pyrimidine synthesis catalyzed by the mitochondrial enzyme dihydroorotate dehydrogenase (DHOD).

Serotonin and tryptophan metabolism

Serotonin was decreased in both males and females, but its precursor tryptophan, was unchanged in this cohort of drug-free rrMDD subjects (Figure 2G).

Phenylalanine and tyrosine metabolism

Phenylketones is the name given to molecules made largely by gut bacteria from phenylalanine and tyrosine left over after, or diverted from, catecholamine neurotransmitter and protein synthesis. Phenyllactic acid is a phenylketone that was decreased in females. Hydroxyphenylacetic acid is a phenylketone that was decreased in males with rrMDD (Figure 2G, Table S3, S4).

SUPPLEMENTARY DISCUSSION

Study Limitations

A small but significant difference was found in some participant characteristics among the female subjects (Table 1). These included a small increase in waist circumference and the number of over-the-counter (OTC) supplements, vitamins, and non-CNS prescription medications. We chose not to correct for these differences statistically for two reasons. First, in the case of waist circumference, we and others have found that this and other measures of obesity such as body mass index (BMI) are intrinsically linked to disturbances in mitochondrial function leading to oxidative stress[15], and as such is not a confounder of the metabolomic signature, but a causal factor or mediator. Statistical efforts to correct for weight can inadvertently over-correct for a mediator of the metabolic phenotype and lead to an increase in type II error. Second, in the case of non-CNS prescription medications, no more than 1-4 patients (2%-10%) were taking any particular drug. As only a minority of subjects was found to take any particular medication, we felt the chances of introducing an error by inadvertent over-correction or over-simplification by grouping different types of drugs by category, were greater than the potential benefit of correcting for a factor that was present in only a minority of the subjects. Larger studies will be required to sort out the effects of non-CNS medications on the metabolomic features of recurrent major depressive disorder.

Mechanistic Implications

Lipids dominated both the diagnostic and prognostic metabolic markers found in rrMDD. Purine abnormalities were also a consistent feature in both diagnostic and prognostic markers. The importance of lipids and purines in major depressive disorder [16], anxiety [17], autism spectrum disorder [18], and other mental health disorders [19,20] has been underscored by several recent studies.

The prognostic metabolites that were found to regulate the risk of recurrence were united in serving dual functions in the cell: 1. as matter, these molecules function as building blocks and intermediates in metabolism; 2. as information, they can act to modify macromolecular targets and change their function by phosphorylation, methylation, acetylation, myristoylation, farnesylation, etc. In addition, these metabolites act as ligands for transmembrane G-protein coupled or nuclear receptors that

change gene expression, or act as allosteric regulators that change the conductance of solute carriers (SLCs) and ATP-binding cassette (ABC) transporters that conduct metabolites as organic anions and cations, and ion channels that conduct inorganic cations like Na^+, K^+, Mg^{2+}, and Ca^{2+}, and anions like Cl^-. In the case of purines, a third function is also well known: the role of molecules like ATP as energy. Metabolites that have both metabolic and signaling functions have been called "metabokines". All neurotransmitters currently targeted by drug therapies for major depressive disorder are metabokines [21], and purines are purinergic metabokines that act as ligands for ionotropic and metabotropic receptors [22,23]. A large majority of metabokines have been found to be coordinately regulated in response to cellular injury or threat. The coordinated regulation of metabokines has been placed in evolutionary context as the biological process that underlies healing and aging and called the cell danger response (CDR) [21,24,25]. The molecular aspects of the CDR include the integrated stress response (ISR)[26]. The long-distance coordination of the CDR is effected by metabokines used for remote sensing and signaling (RSS) [27]. The interconversion of these 3 functions of metabolites as matter, information, and energy requires the movement of molecules up and down gradients between membrane-bounded compartments—between organelles, between cells, the extracellular space, and between organ systems—such that information is stored in the form of dynamically-interacting structures acting as sub-systems in the larger whole-body system. The subsystems of the body are connected and coordinated by the flow of materials they exchange [28-30], the energy they produce [31,32], and the information they store as structural order [33,34] and epigenetic modifications [35,36].

Potential Clinical Implications for Prognosis, Management and Treatment

Low methylcysteine was the single most predictive risk factor for recurrence found in females with recurrent major depression in remission. Low methylcysteine has been found to be a risk factor for a number of other mental health disorders including schizophrenia [37,38]. Dietary sources of methylcysteine include onions, garlic, and cruciferous vegetables like cabbage, broccoli, and kale and many legumes like peas, soy, and kidney beans [39]. Recent clinical trials [40,41] have found that dietary interventions can increase plasma methylcysteine levels, in addition to several sphingolipids and

phospholipids. However, methylcysteine alone accounted for only 6% of the risk of recurrence in females and was not a predictor of recurrence in males. The broader benefits of tailored healthy dietary interventions should be considered as a potential intervention for patients with recurrent major depressive disorder. Low phospholipid and sphingolipids, which can also be increased by including a healthy diet, together added another 47% of the recurrence risk in females and 68% in males. The importance of sphingolipids in depression has been underscored by the recent demonstration that a common mechanism of action for many antidepressant drugs is to inhibit the stress-related conversion of sphingomyelin to ceramide by inhibiting acid sphingomyelinase (ASM), thereby restoring more normal sphingomyelin levels, autophagy, and organellar quality control[42]. Abnormalities in metabolites traceable to the microbiome also accounted for 6% of the recurrence risk in both males and females. Although low methylcysteine was the top predictor of recurrence in females, a decrease in 10 sphingolipids and 5 phospholipids was responsible for a larger proportion of the overall metabolic risk. Low 15-HETE, β-carotene, and 7 sphingolipids were the top predictors in males.

If methylcysteine, phospholipids, sphingolipids (ceramides, sphingomyelins, glycosphingolipids), β-carotene, and the microbiome are addressed together, more than 80% of the metabolic risk of recurrence might be amenable to intervention. However, vitamin supplementation alone is not a solution. Accumulating evidence suggests that over-supplementation with purified vitamins can distort their natural stoichiometric proportions *in vivo.* Vitamin ratio distortions can produce relative deficiencies in unsupplemented vitamins because balanced proportions in vitamin cofactors are required to maintain balanced metabolic fluxes. Improvements in weekly activity and exercise can be used to facilitate the whole body integration of metabolism, and improve brain-body and interorgan communication, mitochondrial quality control by mitophagy [43], lipid, endocrine, inflammation, and microbiome health [44-46]. Clinical trials of a whole food diet rich in plant-based foods containing methylcysteine, carotenoids, phospholipids, sphingolipids, and fiber for a healthier microbiome, with

and without judicious supplementation in selected subjects, and exercise, could be a natural next step in the application of the results of this metabolomic study.

SUPPLEMENTARY REFERENCES

1. Xia, J., Sinelnikov, I.V., Han, B. & Wishart, D.S. MetaboAnalyst 3.0-making metabolomics more meaningful. *Nucleic acids research* **43**, W251-W257 (2015).
2. Chong, J.*, et al.* MetaboAnalyst 4.0: towards more transparent and integrative metabolomics analysis. *Nucleic acids research* (2018).
3. Benjamini, Y. & Hochberg, Y. Controlling the false discovery rate—a practical and powerful approach to multiple testing. *Journal of the Royal Statistical Society Series B-Methodological* **57**, 289-300 (1995).
4. Storey, J.D. The positive false discovery rate: a Bayesian interpretation and the q-value. *The Annals of Statistics* **31**, 2013-2035 (2003).
5. Breiman, L. Random Forests. *Machine Learning* **45**, 5-32 (2001).
6. Fisher, L.D. & Lin, D.Y. Time-dependent covariates in the Cox proportional-hazards regression model. *Annu Rev Public Health* **20**, 145-157 (1999).
7. Figueroa, C.A.*, et al.* Cognitive reactivity versus dysfunctional cognitions and the prediction of relapse in recurrent major depressive disorder. *The Journal of clinical psychiatry* **76**, e1306-1312 (2015).
8. Filzmoser, P., Liebmann, B. & Varmuza, K. Repeated double cross validation. *J Chemometrics* **23**, 160-171 (2009).
9. Szymanska, E., Saccenti, E., Smilde, A.K. & Westerhuis, J.A. Double-check: validation of diagnostic statistics for PLS-DA models in metabolomics studies. *Metabolomics : Official journal of the Metabolomic Society* **8**, 3-16 (2012).
10. Zheng, P.*, et al.* Identification of sex-specific urinary biomarkers for major depressive disorder by combined application of NMR- and GC-MS-based metabonomics. *Translational psychiatry* **6**, e955 (2016).
11. Li, M.Y.*, et al.* Antineoplastic effects of 15(S)-hydroxyeicosatetraenoic acid and 13-S-hydroxyoctadecadienoic acid in non-small cell lung cancer. *Cancer* **121 Suppl 17**, 3130-3145 (2015).
12. Powell, W.S., Gravelle, F. & Gravel, S. Metabolism of 5(S)-hydroxy-6,8,11,14-eicosatetraenoic acid and other 5(S)-hydroxyeicosanoids by a specific dehydrogenase in human polymorphonuclear leukocytes. *The Journal of biological chemistry* **267**, 19233-19241 (1992).
13. Goetzl, E.J., Weller, P.F. & Sun, F.F. The regulation of human eosinophil function by endogenous mono-hydroxy-eicosatetraenoic acids (HETEs). *J Immunol* **124**, 926-933 (1980).
14. Wang, Y.*, et al.* Metabolomic analysis of serum from obese adults with hyperlipemia by UHPLC-Q-TOF MS/MS. *Biomed Chromatogr* **30**, 48-54 (2016).
15. Assies, J.*, et al.* Effects of oxidative stress on fatty acid- and one-carbon-metabolism in psychiatric and cardiovascular disease comorbidity. *Acta psychiatrica Scandinavica* **130**, 163-180 (2014).
16. Zhou, X.*, et al.* Polyunsaturated fatty acids metabolism, purine metabolism and inosine as potential independent diagnostic biomarkers for major depressive disorder in children and adolescents. *Molecular psychiatry* **24**, 1478-1488 (2019).
17. Fan, K.Q.*, et al.* Stress-Induced Metabolic Disorder in Peripheral CD4(+) T Cells Leads to Anxiety-like Behavior. *Cell* **179**, 864-879 e819 (2019).
18. Naviaux, R.K.*, et al.* Low-dose suramin in autism spectrum disorder: a small, phase I/II, randomized clinical trial. *Ann Clin Transl Neurol* **4**, 491-505 (2017).
19. Krugel, U. Purinergic receptors in psychiatric disorders. *Neuropharmacology* **104**, 212-225 (2016).
20. Schneider, M.*, et al.* Lipids in psychiatric disorders and preventive medicine. *Neuroscience and biobehavioral reviews* **76**, 336-362 (2017).
21. Naviaux, R.K. Metabolic features and regulation of the healing cycle-A new model for chronic disease pathogenesis and treatment. *Mitochondrion* **46**, 278-297 (2019).
22. Cheffer, A.*, et al.* Purinergic system in psychiatric diseases. *Molecular psychiatry* **23**, 94-106 (2018).

23. Burnstock, G. The therapeutic potential of purinergic signalling. *Biochemical pharmacology* **151**, 157-165 (2018).
24. Naviaux, R.K. Metabolic features of the cell danger response. *Mitochondrion* **16**, 7-17 (2014).
25. Naviaux, R.K. Incomplete Healing as a Cause of Aging: The Role of Mitochondria and the Cell Danger Response. *Biology (Basel)* **8**(2019).
26. Pakos-Zebrucka, K.*, et al.* The integrated stress response. *EMBO Rep* **17**, 1374-1395 (2016).
27. Nigam, S.K. What do drug transporters really do? *Nature reviews. Drug discovery* **14**, 29-44 (2015).
28. Kondepudi, D. & Prigogine, I. *Modern thermodynamics: from heat engines to dissipative structures*, (John Wiley & Sons, 2014).
29. Prigogine, I. & Nicolis, G. Biological order, structure and instabilities. *Q Rev Biophys* **4**, 107-148 (1971).
30. Prigogine, I. & Lefever, R. Symmetry breaking instabilities in dissipative systems. II. *The Journal of Chemical Physics* **48**, 1695-1700 (1968).
31. Wallace, D.C. Bioenergetic origins of complexity and disease. *Cold Spring Harbor symposia on quantitative biology* **76**, 1-16 (2011).
32. Wallace, D.C. A mitochondrial bioenergetic etiology of disease. *The Journal of clinical investigation* **123**, 1405-1412 (2013).
33. Pollack, G.H. *The Fourth Phase of Water: Beyond Solid, Liquid, and Vapor*, (Ebner & Sons Seattle, WA, 2013).
34. Cunliffe, J. Tissue homeostasis and immunity--more on models. *Scandinavian journal of immunology* **64**, 172-176 (2006).
35. Wallace, D.C. Bioenergetics and the epigenome: interface between the environment and genes in common diseases. *Developmental disabilities research reviews* **16**, 114-119 (2010).
36. Smiraglia, D.J., Kulawiec, M., Bistulfi, G.L., Gupta, S.G. & Singh, K.K. A novel role for mitochondria in regulating epigenetic modification in the nucleus. *Cancer biology & therapy* **7**, 1182-1190 (2008).
37. Cao, B.*, et al.* Characterizing amino-acid biosignatures amongst individuals with schizophrenia: a case-control study. *Amino acids* **50**, 1013-1023 (2018).
38. Guo, Y.*, et al.* Preoperative Serum Metabolites Are Associated With Postoperative Delirium in Elderly Hip-Fracture Patients. *J Gerontol A Biol Sci Med Sci* **72**, 1689-1696 (2017).
39. Maw, G.A. Biochemistry of S-methyl-L-cysteine and its principal derivatives. *Sulfur Reports* **2**, 1-26 (1982).
40. Li, K.J., Borresen, E.C., Jenkins-Puccetti, N., Luckasen, G. & Ryan, E.P. Navy Bean and Rice Bran Intake Alters the Plasma Metabolome of Children at Risk for Cardiovascular Disease. *Front Nutr* **4**, 71 (2017).
41. Sheflin, A.M.*, et al.* Dietary supplementation with rice bran or navy bean alters gut bacterial metabolism in colorectal cancer survivors. *Molecular nutrition & food research* **61**(2017).
42. Gulbins, A.*, et al.* Antidepressants act by inducing autophagy controlled by sphingomyelin-ceramide. *Molecular psychiatry* (2018).
43. Kim, Y., Triolo, M. & Hood, D.A. Impact of Aging and Exercise on Mitochondrial Quality Control in Skeletal Muscle. *Oxidative medicine and cellular longevity* **2017**, 3165396 (2017).
44. Farioli Vecchioli, S., Sacchetti, S., Nicolis di Robilant, V. & Cutuli, D. The Role of Physical Exercise and Omega-3 Fatty Acids in Depressive Illness in the Elderly. *Curr Neuropharmacol* **16**, 308-326 (2018).
45. Yu, H.J.*, et al.* Improving the Metabolic and Mental Health of Children with Obesity: A School-Based Nutrition Education and Physical Activity Intervention in Wuhan, China. *Nutrients* **12**(2020).
46. Ignacio, Z.M., da Silva, R.S., Plissari, M.E., Quevedo, J. & Reus, G.Z. Physical Exercise and Neuroinflammation in Major Depressive Disorder. *Molecular neurobiology* **56**, 8323-8335 (2019).

Single PDF

Supplementary Methods, Results, and Discussion.

Figure S1. Principal component analysis of metabolomics in rrMDD. **A.** Diagnostic differences, rrMDD vs healthy controls (n = 84 females). **B.** Prognostic differences, prospective analysis of rrMDD subjects with recurrence and non-recurrence in 2.5 years (n = 42 females). **C.** Diagnostic differences, rrMDD vs healthy controls (n = 42 males). **D.** Prognostic differences, prospective analysis of rrMDD subjects with recurrence and non-recurrence in 2.5 years (n = 20 males). The green lines indicate the cumulative proportion of phenotypic variation explained by up to 5 principal components. The blue lines indicate the fraction of variance explained by each component.

Figure S2. Cytoscape map of metabolic pathways altered in rrMDD compared to healthy controls, **A.** Females, **B.** Males. rrMDD subjects *n* = 44 females, 23 males. Controls *n* = 40 females, 19 males.

Figure S3. Shared and gender-specific metabolic differences that distinguish subjects with recurrent major depressive disorder in remission from controls.

Figure S4. Kaplan-Meier style recurrence curves for the top metabolic predictors of recurrence risk in females with a history of recurrent major depressive disorder in drug-free remission identified by Cox proportional hazard analysis. Subjects were stratified by risk observed in the top vs the bottom 50th percentile for each metabolite.

Figure S5. Kaplan-Meier style recurrence curves for the top metabolic predictors of recurrence risk in males with a history of recurrent major depressive disorder in drug-free remission identified by Cox proportional hazard analysis. Subjects were stratified by risk observed in the top vs the bottom 50th percentile for each metabolite.

Single Excel File

Table S1. Raw AUC and recurrence data. Females
Table S2. Raw AUC and recurrence data. Males
Table S3. Biochemical pathways disturbed with recurrent major depressive disorder in remission: Females.
Table S4. Biochemical pathways disturbed with recurrent major depressive disorder in remission: Males.
Table S5. Statistical analysis. Females
Table S6. Statistical analysis. Males
Table S7. Prognostic pathways. Females
Table S8. Prognostic pathways. Males
Table S9. Representative diagnostic and prognostic metabolites for recurrent major depressive disorder in remission and the risk of recurrence. Females
Table S10. Representative diagnostic and prognostic metabolites for recurrent major depressive disorder in remission and the risk of recurrence. Males
Table S11. Log transformations. Females
Table S12. Z-score transformations. Females
Table S13. Log transformations. Males
Table S14. Z-score transformations. Males

Supplementary Figures and Legends

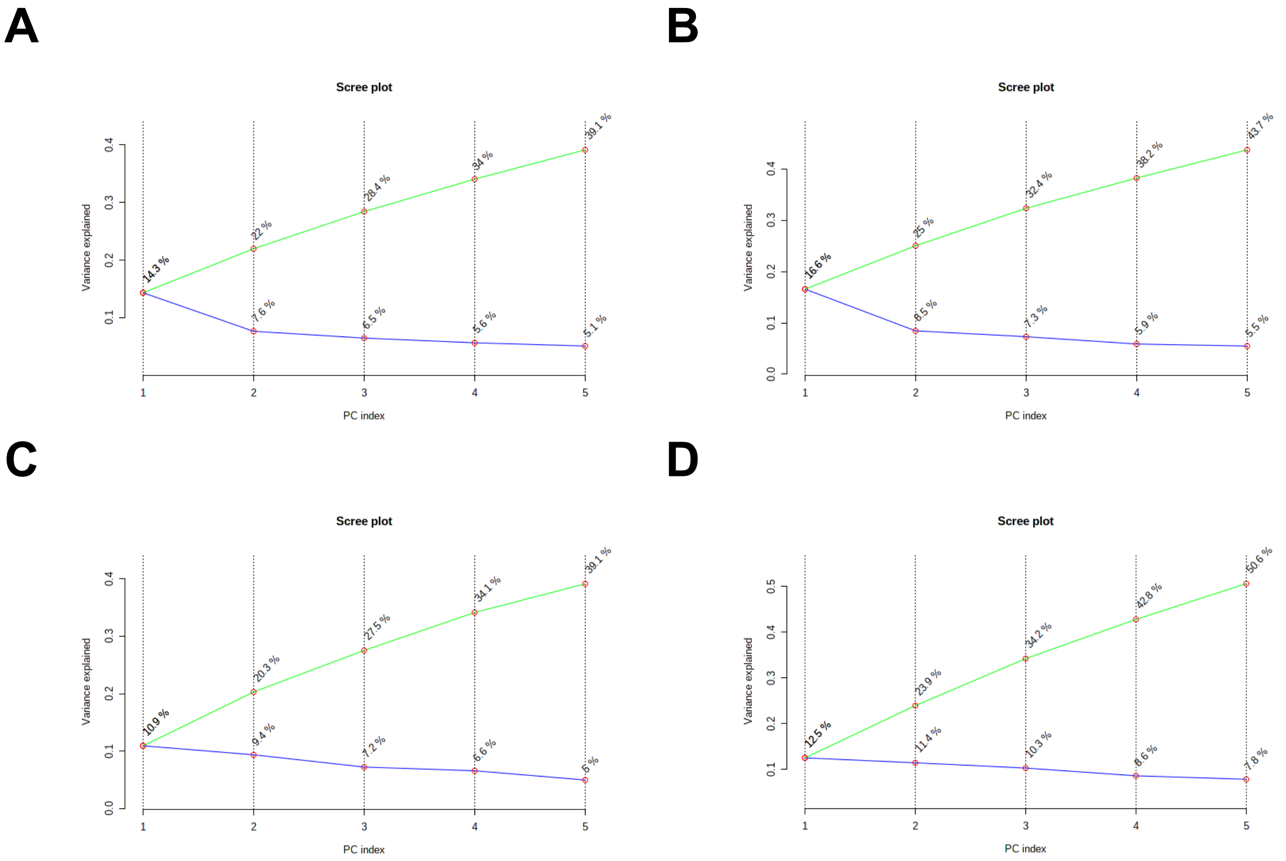

Figure S1. Principal component analysis of metabolomics in rrMDD. **A.** Diagnostic differences, rrMDD vs healthy controls (n = 84 females). **B.** Prognostic differences, prospective analysis of rrMDD subjects with recurrence and non-recurrence in 2.5 years (n = 42 females). **C.** Diagnostic differences, rrMDD vs healthy controls (n = 42 males). **D.** Prognostic differences, prospective analysis of rrMDD subjects with recurrence and non-recurrence in 2.5 years (n = 20 males). The green lines indicate the cumulative proportion of phenotypic variation explained by up to 5 principal components. The blue lines indicate the fraction of variance explained by each component.

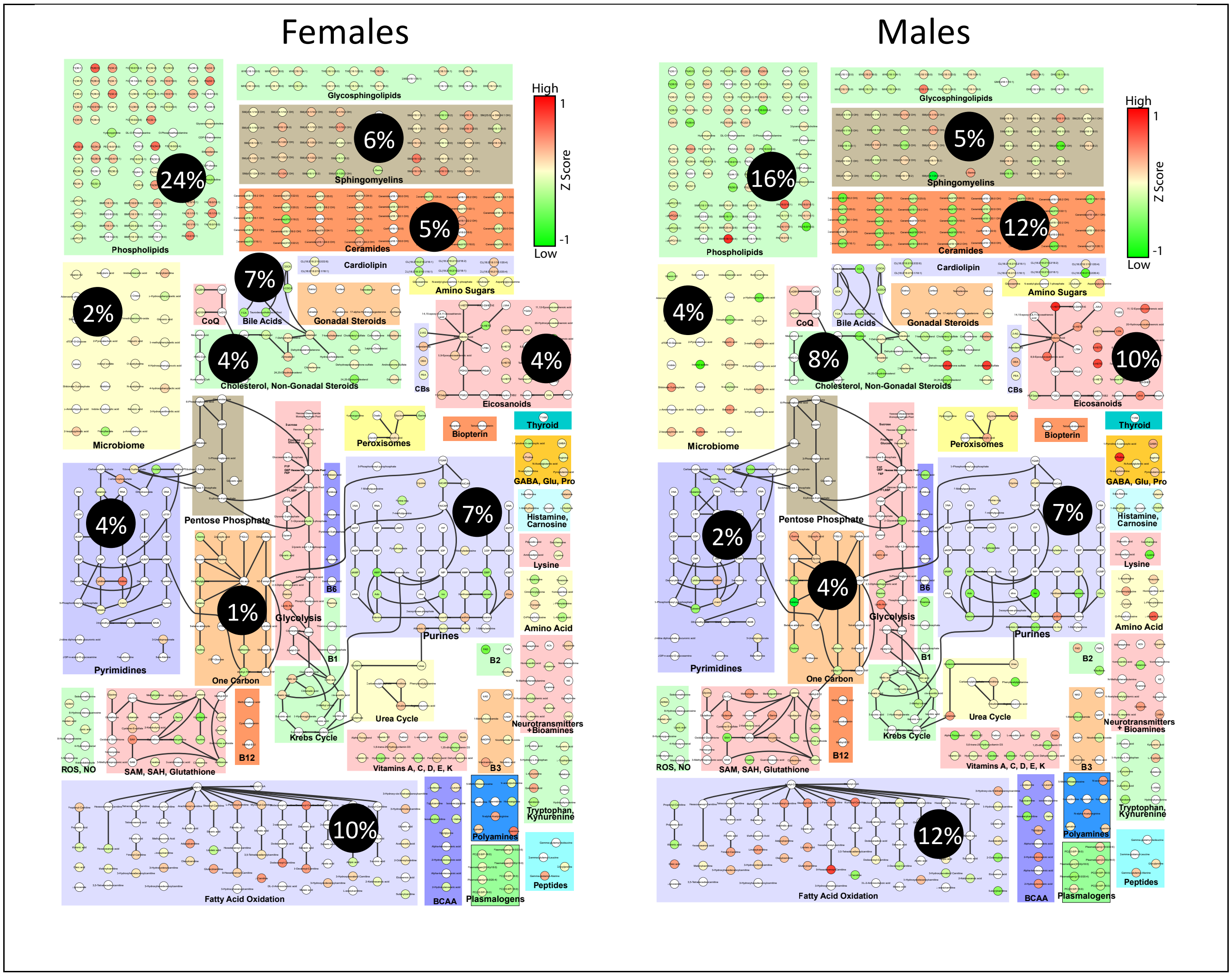

Figure S2. Cytoscape map of metabolic pathways altered in rrMDD compared to healthy controls, **A.** Females, **B.** Males. rrMDD subjects *n* = 44 females, 23 males. Controls *n* = 40 females, 19 males.

A **B**

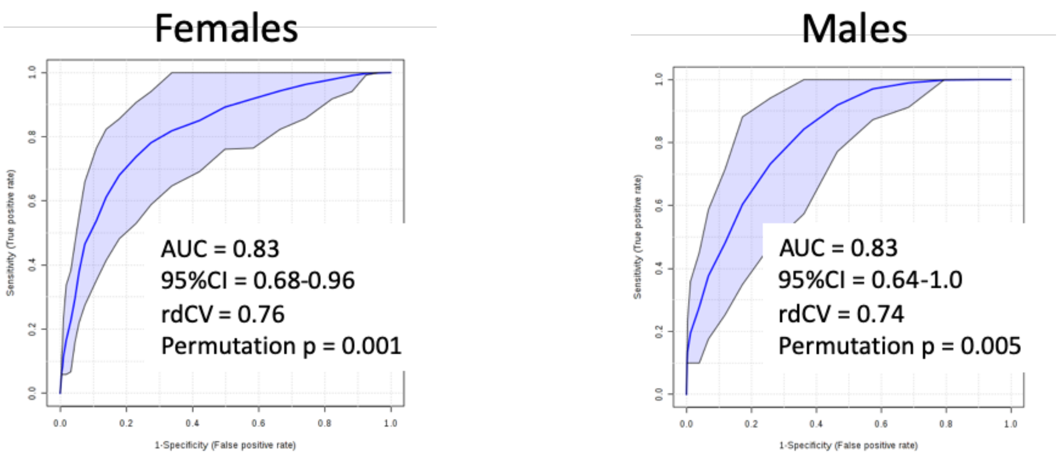

C

↓ 13(S)-HODE
↓ FAD
↓ AMP
↓ Hypotaurine
↑ Quinolinic acid
↑ PG(34:2)
↓ 24,25-Epoxycholesterol
↓ CL(18:2/18:2/18:2/18:1)
↓ Phenyllactic acid
↑ Octanoylcarnitine
↑ Decanoylcarnitine
↑ Proline

	RMDD-IR	Control
Positive	35	6
Negative	9	34

Sensitivity = 0.80 (95% CI = 0.66-0.89)
Specificity = 0.87 (95% CI = 0.74-0.95)

D

↓ SM(d18:1/26:0 OH)
↑ DHEA-S
↑ 11-HETE
↓ CL(18:2/18:2/18:2/20:4)
↓ Lathosterol
↓ Betaine
↓ SM(d18:1/26:2 OH)

	RMDD-IR	Control
Positive	17	4
Negative	6	15

Sensitivity = 0.74 (95% CI = 0.53-0.87)
Specificity = 0.79 (95% CI = 0.57-0.91)

Figure S3. Shared and gender-specific metabolic differences that distinguish subjects with recurrent major depressive disorder in remission from controls. Females: **A** and **C.** Males: **B** and **D. AB** Receiver operator characteristic (ROC) curve analysis of multianalyte diagnostic classifiers for rrMDD. The classifier for females used 12 metabolites. The classifier for males used 7 metabolites. AUC: area under the curve; rdCV: repeated double cross validation accuracy. **CD.** 2 x 2 contingency table analysis. rrMDD subjects *n* = 44 females, 23 males. Controls *n* = 40 females, 19 males.

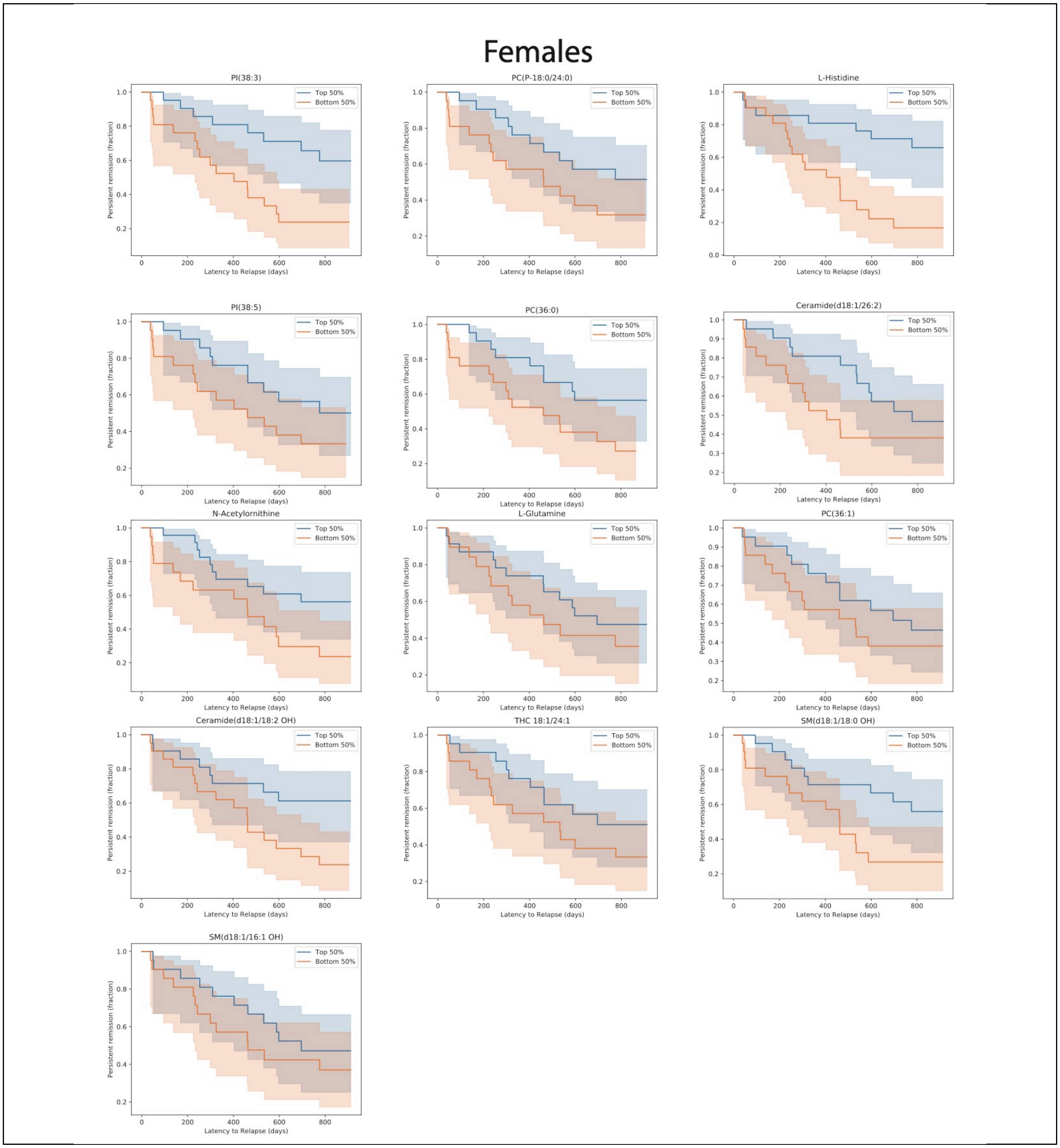

Figure S4. Kaplan-Meier style recurrence curves for the top metabolic predictors of recurrence risk in females with a history of recurrent major depressive disorder in drug-free remission identified by Cox proportional hazard analysis. rrMDD subjects were followed prospectively for 2.5 years: n = 42 females (24 with recurrence, 18 no recurrence).

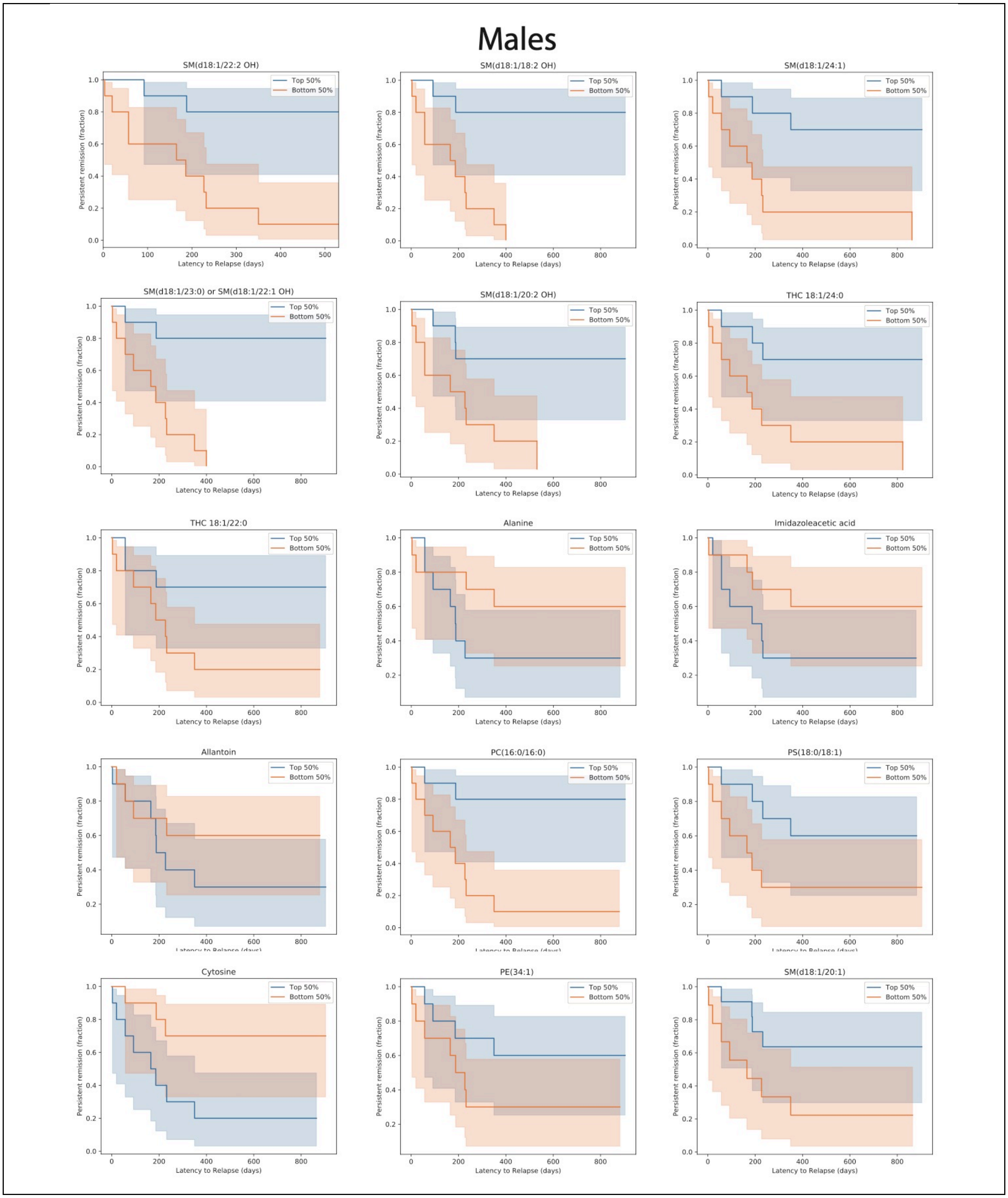

Figure S5. Kaplan-Meier style recurrence curves for the top metabolic predictors of recurrence risk in males with a history of recurrent major depressive disorder in drug-free remission identified by Cox proportional hazard analysis. rrMDD subjects were followed prospectively for 2.5 years: *n* = 20 males (11 with recurrence, 9 no recurrence).
